# The Impact of Virtual Reality Content Characteristics on Cybersickness and Head Movement Patterns

**DOI:** 10.3390/s25010215

**Published:** 2025-01-02

**Authors:** Seo-Yoon Park, Dong-Kyun Koo

**Affiliations:** 1 Department of Physical Therapy, College of Health and Welfare, Woosuk University, 443 Samnye-ro, Samnye-eup, Wanju-gun 55338, Republic of Korea; pgy0614@hanmail.net; 2 University-Industrial Cooperation Corps of HiVE Center, Wonkwang Health Science University, 514, Iksan-daero, Iksan-si 54538, Republic of Korea

**Keywords:** virtual reality, cybersickness, head movement patterns, sensory conflict, movement analysis, visual–vestibular interaction

## Abstract

Virtual reality (VR) technology has gained popularity across various fields; however, its use often induces cybersickness, characterized by symptoms such as dizziness, nausea, and eye strain. This study investigated the differences in cybersickness levels and head movement patterns under three distinct VR viewing conditions: dynamic VR (DVR), static VR (SVR), and a control condition (CON) using a simulator. Thirty healthy adults participated, and their head movements were recorded using the Meta Quest 2 VR headset and analyzed using Python. The Virtual Reality Sickness Questionnaire (VRSQ) assessed subjective cybersickness levels. The results revealed that the SVR condition induced the highest VRSQ scores (M = 58.057), indicating the most severe cybersickness symptoms, while the DVR condition elicited significantly higher values in head movement variables, particularly in the coefficient of variation (CV) and integral values of head position along the vertical axis, and mean velocity (*p* < 0.05). These findings suggest that VR content characteristics directly influence users’ head movement patterns, closely related to cybersickness occurrence and severity. This study highlights the importance of analyzing head movement patterns in cybersickness research and provides insights for VR content design.

## 1. Introduction

Virtual reality (VR) is a technology that utilizes computer graphics to create a simulated 3D environment, providing users with a sense of immersion and presence [1]. VR technology has been applied in various fields such as gaming, education, medicine, and training, and is evolving rapidly [2,3]. However, cybersickness, a condition similar to motion sickness, can occur during VR viewing, negatively impacting user experience [4]. Despite significant technological advances in hardware and software over the past decade, cybersickness remains a persistent challenge affecting 40–70% of users, limiting VR’s widespread adoption across various sectors, including healthcare, education, and entertainment [5,6].

Cybersickness is characterized by symptoms such as dizziness, nausea, headache, and eye strain, which are experienced while viewing VR content [7]. While modern VR headsets with improved refresh rates and motion tracking have reduced these symptoms compared to earlier technologies, cybersickness continues to impact user experience and practical applications [8]. These symptoms are thought to be caused by factors such as sensory conflict between visual and vestibular information, system latency, and individual differences in susceptibility. Cybersickness can reduce users’ sense of immersion and presence, limiting the widespread adoption of VR technology [9,10].

Previous studies have indicated a close relationship between head movements and cybersickness during VR viewing. Kim et al. [11] measured head movements and assessed the degree of cybersickness while participants viewed a dynamic virtual environment using a head-mounted display (HMD). The results showed that the group experiencing cybersickness had significantly larger head movement amplitudes compared to the group not experiencing cybersickness.

Additionally, Palmisano et al. [12] analyzed the correlation between head movements and cybersickness while participants viewed various virtual environments using an HMD VR device. The results revealed that head movement patterns differed depending on the type of virtual environment, and this had a significant correlation with the degree of cybersickness. Although these prior studies suggested a link between head movements and cybersickness, they primarily focused on analyzing differences in head movements and cybersickness based on the type of virtual environment [13,14]. There is a need for a more comprehensive and detailed quantitative analysis of head motion patterns during VR viewing to better elucidate the relationship between head movements and cybersickness.

The present study aims to address this gap by establishing three VR viewing conditions (groups) and conducting a thorough analysis of head motion patterns in each condition. By employing a comprehensive set of variables, including the mean; coefficient of variation; and integral of head position, orientation, and speed, this study provides a detailed quantitative examination of head motion characteristics during VR viewing. This in-depth analysis distinguishes our research from previous studies and contributes to a better understanding of the relationship between head movements and cybersickness [15].

Furthermore, by comprehensively investigating the influence of VR viewing conditions on cybersickness and head movements, this research seeks to contribute to the development of evidence-based strategies for minimizing cybersickness. The findings of this study can potentially inform the design of VR systems and applications that reduce the incidence and severity of cybersickness, ultimately enhancing user experience and the usability of VR technology.

## 2. Materials and Methods

### 2.1. Participants

The required sample size was determined a priori using G*Power software (version 3.1.9.7). With an effect size (f) of 0.25, a significance level (α) of 0.05, and a power (1 − β) of 0.8, assuming three measurement points and a correlation of 0.5 between repeated measures in a repeated measures ANOVA, the minimum required sample size was calculated to be 30 participants [16]. Based on this, open recruitment was conducted targeting healthy adults in their 20s to ensure a sufficient number of participants. A total of 30 participants (15 males, 15 females) were included in the study, with a mean age of 25.95 ± 4.02 years, height of 169.20 ± 7.22 cm, and weight of 64.40 ± 13.73 kg. All demographic data were collected through self-report questionnaires. However, two participants who experienced severe dizziness were excluded from the study.

The inclusion criteria for the study participants were as follows: (1) healthy male and female adults in their 20s, (2) physically and mentally healthy individuals, and (3) those who understood the study’s purpose and procedures and voluntarily agreed to participate. The exclusion criteria were as follows: (1) individuals who had used VR more than once a month, (2) those with severe eye or vestibular disorders, (3) pregnant or lactating women, and (4) those with physical or mental disorders that may affect their participation in the study.

### 2.2. Materials

The Meta Quest 2 (Meta Platforms, Inc., USA, originally sold as Oculus Quest 2, Facebook Technologies, LLC, USA) VR headset was used in this study. The Meta Quest 2 is a wireless standalone VR headset with a resolution of 1832 × 1920 pixels per eye and supports a maximum refresh rate of 120 Hz [17]. It also provides 6-degree-of-freedom (6DOF) motion tracking, allowing accurate tracking of the user’s position and orientation.

**Figure 1:**
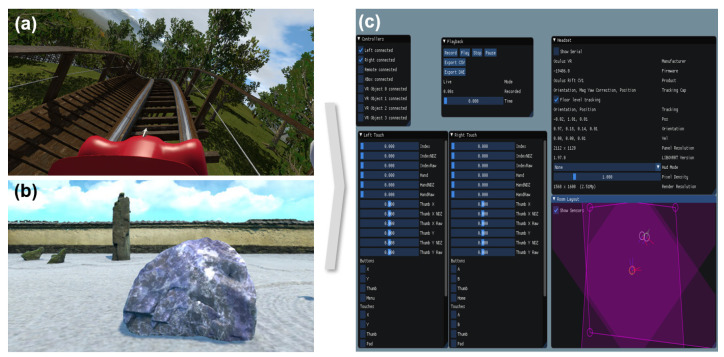
Oculus VR program and measurement software displays. (**a**) NoLimits 2 roller coaster simulation used in dynamic/static VR condition. (**b**) Rock Simulator used in static VR condition (control condition). (**c**) Oculus Monitor software interface for recording and monitoring head tracking data (position, orientation, velocity) in real time during VR use.

NoLimits 2 (Mad Data GmbH, Erkrath, Germany) was used in both the dynamic VR (DVR) and static VR (SVR) conditions. NoLimits 2 is a roller coaster simulation that offers a realistic physics engine and high-quality graphics, and is also used in the design of actual roller coasters (Figure 1a). In the control condition (CON), Rock Simulator was used. Rock Simulator is a static VR environment that provides an experience of being a rock and observing the passage of time (Figure 1b).

To collect head tracking data from the participants, the Oculus Monitor [18] software was used. Oculus Monitor is a tool that can record and monitor the headset’s position, orientation, velocity, and other data in real time during VR use (Figure 1c). The VRSQ was used for the subjective assessment of VR cybersickness. According to Kim et al. [11], the VRSQ demonstrated high internal consistency (Cronbach’s α = 0.92) and test–retest reliability (r = 0.80, *p* < 0.001). Convergent validity was also demonstrated through a high correlation (r = 0.78, *p* < 0.001) between the VRSQ scores and the Simulator Sickness Questionnaire (SSQ) scores.

### 2.3. Experimental Procedure

**Figure 2:**
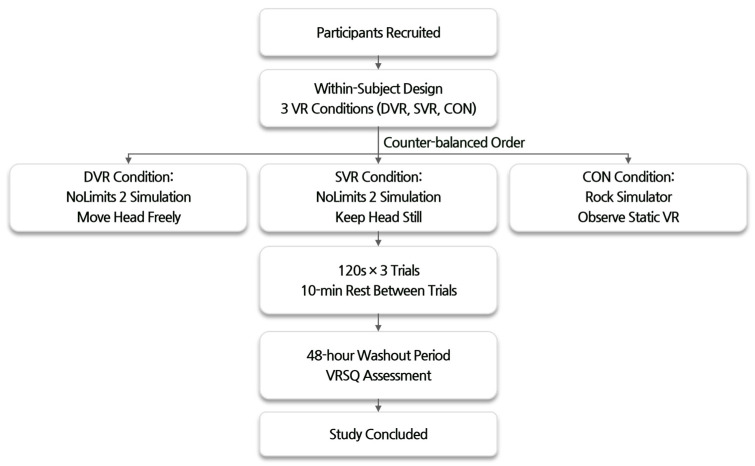
A flow diagram of the experimental design and procedure. The study employed a within-subject design with three VR conditions (DVR: dynamic VR; SVR: static VR; CON: control) administered in a counter-balanced order. Each condition consisted of three 120 s trials with 10 min rest periods between trials. A 48 h washout period was implemented between conditions to minimize potential carryover effects. VRSQ assessments were conducted after each condition to evaluate cybersickness symptoms.

The experiment was conducted using a within-subject design, and the participants took part in the three VR viewing conditions (DVR, SVR, and CON) in a counterbalanced order (Figure 2).

In the DVR condition, participants watched the NoLimits 2 roller coaster simulation and were instructed to move their heads freely and naturally in response to the motion of the virtual roller coaster. In the SVR condition, participants watched the same roller coaster simulation but were told to keep their heads as still as possible while focusing on the virtual content. In the CON condition, participants watched the Rock Simulator, which provided a static VR environment where they were asked to observe the virtual environment without any specific head movement instructions.

Each VR condition lasted for 120 s, and participants wore the Meta Quest 2 VR headset during the DVR and SVR conditions. Each condition was measured three times, with a 10 min rest period between repeated measurements to minimize learning effects. This rest period was based on previous research reporting that a 10 min break after VR exposure is sufficient to alleviate cybersickness symptoms and prepare for the next VR trial [19].

A 48 h washout period was implemented between each VR condition to offset any potential VR adaptation effects [20]. Immediately after each VR condition, the participants completed the VRSQ to assess their subjective levels of cybersickness. After all conditions were completed, the participants were compensated for their participation, and the experiment was concluded. This study was approved by the Institutional Review Board (IRB) of Dankook University, Republic of Korea (IRB No. DKU 2021-03-069).

### 2.4. Data Collection and Processing

During the experiment, the Oculus Monitor software was used to record the participants’ head tracking data (position, orientation, velocity, etc.). The recorded head tracking data were exported in CSV format and further analyzed using the Python programming language and related libraries (NumPy, Matplotlib, etc.). The data analysis process involved applying a Kalman filter for noise reduction, calculating statistical measures (mean, coefficient of variation, integral value, etc.) for the head position and orientation data, and analyzing velocity data and statistical measures of velocity magnitude.

The VRSQ scores and variables derived from the head tracking data were analyzed using a one-way repeated measures ANOVA. Prior to conducting the one-way repeated measures ANOVA, we tested the normality assumption using the Shapiro–Wilk test for all dependent variables. The results showed that the data were normally distributed (*p* > 0.05) for all variables. Additionally, we verified the sphericity assumption using Mauchly’s test. When the assumption of sphericity was violated, the Greenhouse–Geisser correction was applied. Tukey’s HSD test was used for post hoc comparisons. The significance level was set at α = 0.05, and all statistical analyses were performed using SPSS 26.0 (IBM Corp., Armonk, NY, USA) software.

## 3. Results

A one-way repeated measures ANOVA was conducted to compare the effects of different VR viewing conditions (DVR, SVR, and CON) on VRSQ scores and head movement variables. Post hoc analyses using Tukey’s HSD test were performed for pairwise comparisons. The significance level was set at alpha = 0.05. All participants’ data were included in the final analysis, with no exclusions or missing data.

The ANOVA revealed a significant main effect of VR condition on VRSQ scores (F(2, 18) = 83.687, *p* < 0.001). Post hoc tests showed that the SVR condition (M = 58.057, SD = 6.364) induced significantly higher VRSQ scores compared to both the DVR (M = 30.557, SD = 10.078, *p* < 0.001) and CON conditions (M = 14.723, SD = 3.851, *p* < 0.001).

Significant main effects of VR condition were also found for several head movement variables (Table 1). The DVR condition elicited significantly higher values than the SVR and CON conditions for the CV (F(2, 18) = 20.000, *p* = 0.008) and integral (F(2, 18) = 687.221, *p* < 0.001) of head position along the Y-axis. Additionally, the DVR condition showed significantly higher mean speed along the X-axis (F(2, 18) = 125.826, *p* < 0.001) and Y-axis (F(2, 18) = 8.180, *p* = 0.039) compared to the SVR and CON conditions.

**Figure 3:**
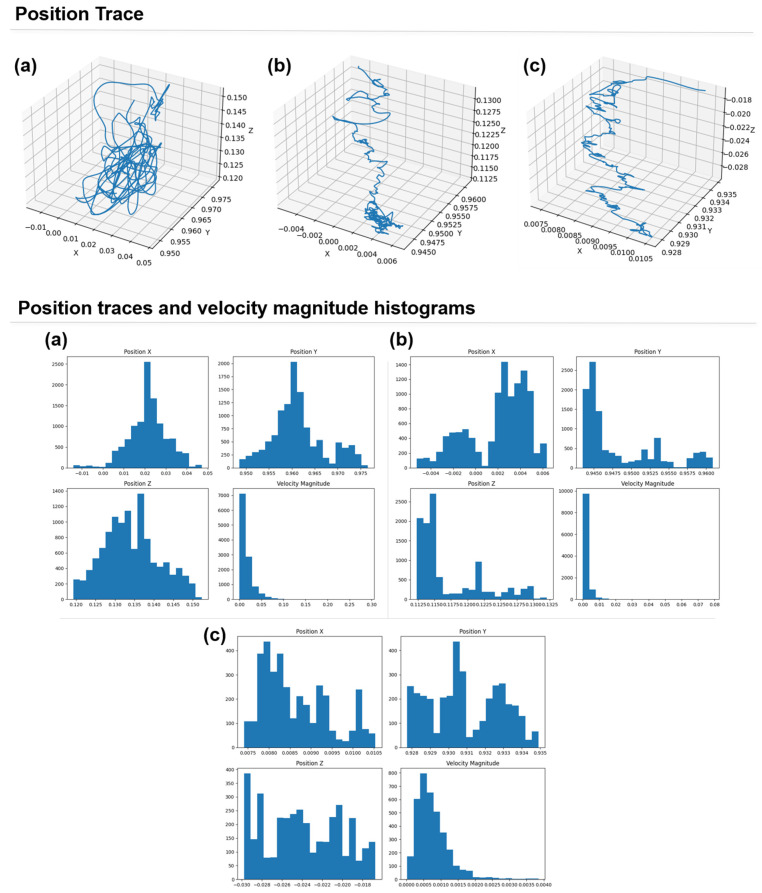
Representative 3D head position traces and corresponding histograms of head position (X, Y, Z axes) and velocity magnitude for a single participant under different VR viewing conditions. (**a**) Dynamic VR condition (DVR): significant head movement in all dimensions, following the VR content motion. (**b**) Static VR condition (SVR): unintentional head movements despite instructions to keep the head still. (**c**) Control condition (CON): stable head position with minimal movement in all dimensions.

The integral of head orientation X (pitch) was significantly higher in the DVR condition compared to the SVR and CON conditions (F(2, 18) = 29.582, *p* = 0.004)(Figure 3). Moreover, the mean head orientation X differed significantly across VR conditions (F(2, 18) = 42.498, *p* = 0.002), with the DVR condition showing the highest values, followed by the SVR and CON conditions.

No significant main effects of VR condition were found for the mean, CV, or integral of head position along the X-axis and Z-axis, or for the mean and integral of head position along the Y-axis (all *p* > 0.05).

Additional effect size calculations (η^2^) revealed that VR conditions had the strongest effect on HeadPosY Integral (η^2^ = 0.987), followed by HeadOrientationW Integral (η^2^ = 0.961) and SpeedX Mean (η^2^ = 0.933). The VRSQ scores also showed a large effect size (η^2^ = 0.903). Post hoc power analysis confirmed excellent statistical power (1 − β > 0.98) for all significant variables, indicating that the sample size was adequate for detecting these effects (Table 2).

## 4. Discussion

The present study aimed to investigate the differences in cybersickness and head movement patterns among various VR viewing conditions. The results showed that the SVR exhibited the highest VRSQ scores, while the DVR demonstrated significantly higher values in the head movement variables, particularly in the position CV, mean velocity, and integral values along the Y-axis.

The higher cybersickness levels in the SVR condition can be attributed to the sensory conflict theory, which posits that a mismatch between visual and vestibular information can lead to cybersickness symptoms. In the SVR, participants experienced visual motion that was incongruent with their head movements, resulting in a sensory conflict. Additionally, the conflict between reflexive head movements driven by the vestibulo-ocular reflex (VOR) and vestibulo-collic reflex (VCR) [21] and the intentional control to keep the head still may have further exacerbated the sensory conflict and cybersickness severity.

Conversely, the significantly higher position CV, mean velocity, and integral values along the Y-axis in the DVR indicate that participants’ head movements were induced by the dynamic vertical motion of the roller coaster VR content. This result demonstrates that the characteristics of the VR content can directly influence users’ head movement patterns [22,23]. Furthermore, the significantly higher mean and integral values of head orientation X (pitch direction) in the DVR highlight the prominence of head nodding (pitch rotation) movements corresponding to the ascents and descents of the roller coaster track.

These findings suggest that the type and characteristics of VR content can lead to distinct head movement patterns, which are closely related to the likelihood of inducing cybersickness [24,25]. In the DVR, head movements along the vertical and pitch directions were actively elicited by the dynamic nature of the content. In contrast, the SVR saw increased variability and range of head movements due to the conflict between intentional control and reflexive movements [26,27].

These differences are linked to the mechanisms of cybersickness. While the sensory conflict theory provides a foundational framework, recent studies employing multimodal analysis have demonstrated that the integration of head movement data with vestibular-ocular responses could provide more precise predictions of cybersickness onset [28]. For example, combining head movement variables with eye-tracking metrics, such as gaze stability and vergence-accommodation patterns, could better explain the sensorimotor adaptations during VR exposure [29,30]. This multimodal approach could particularly help explain why participants in the SVR condition showed higher VRSQ scores despite displaying less head movement [31,32]. 

The results of this study provide important implications for VR content design. Excessively restricting users’ head movements in VR environments may worsen cybersickness; therefore, design strategies should focus on inducing natural head movements and minimizing sensory conflicts [33,34]. This can be achieved by optimizing the visual flow of VR content and improving the synchronization between users’ head movements and visual motion [35,36]. Based on our findings, VR content should be designed to encourage natural head movements that follow the flow of visual elements, as demonstrated in our roller coaster simulation where participants maintained forward gaze and moved naturally with the coaster’s trajectory. For future development, we propose incorporating adaptive field-of-view control and motion platforms to better integrate vestibular function with visual input, which could lead to systems capable of modifying content based on individual user responses and susceptibility to cybersickness.

Moreover, the head movement variables used in this study (mean; CV; and integral values of position, orientation, and velocity) can serve as useful analytical tools in cybersickness research. These variables enable a multi-faceted evaluation of users’ head movement patterns and can help in gaining a deeper understanding of the relationship between VR content characteristics and cybersickness.

This study analyzed the differences in cybersickness levels and head movement patterns under various VR viewing conditions. However, there are several limitations to consider. First, the study was conducted on a relatively small sample of 30 healthy adults in their 20s, which limits the generalizability of our findings. This homogeneous sample may not fully represent the diverse population of VR users across different age groups, particularly considering that VR technology is increasingly being used by people of various ages, from children to elderly adults. Therefore, caution should be exercised when generalizing the results to other age groups or clinical populations. Future research should investigate cybersickness and head movement patterns across diverse age groups, including children, middle-aged adults, and elderly individuals, to better understand how age-related factors might influence cybersickness susceptibility and adaptation. Second, this study utilized only two types of VR content (roller coaster and rock simulators). Consequently, further research is needed to examine cybersickness and head movement patterns in VR content with various genres and characteristics. Third, while this study focused on analyzing head movement data, future research should implement multimodal data analysis by combining (1) head movement kinematics with vestibular–ocular reflex (VOR) measurements to quantify sensory integration efficiency, (2) eye movement parameters (e.g., saccadic movements, fixation stability) synchronized with head motion data to assess gaze stability, and (3) postural sway measurements temporally aligned with head movement data to evaluate overall balance control. Such an integrated analysis could reveal the temporal relationships between different physiological responses during VR exposure and provide more accurate predictors of cybersickness susceptibility. Lastly, this study examined the effects of short-term VR use with an exposure duration of only 2 min, which may not fully capture the cumulative effects and temporal progression of cybersickness symptoms. This brief exposure time might underestimate the severity and complexity of cybersickness that users experience during extended VR sessions. Future longitudinal studies should investigate the impact of prolonged VR use (e.g., 30 min, 1 h, or multiple sessions) on cybersickness and head movement patterns, particularly focusing on (1) the temporal development and progression of cybersickness symptoms, (2) potential adaptation effects over repeated exposures, and (3) the relationship between exposure duration and symptom severity.

## 5. Conclusions

In conclusion, this study contributes to the growing body of research on cybersickness and its relationship with head movement patterns. Our findings highlight the importance of considering VR content characteristics and viewing conditions that minimize the risk of cybersickness when designing VR experiences. Future research could leverage advanced VR headsets with eye-tracking capabilities (e.g., Meta Quest Pro) and cognitive load monitoring (e.g., HP Reverb G2) to provide deeper insights into cybersickness mechanisms. As VR technologies continue to advance and become more widely adopted in various fields, such as education, training, and therapy, understanding the factors that influence cybersickness and developing effective mitigation strategies will be crucial for ensuring the safety, comfort, and enjoyment of VR users. By providing insights into the mechanisms underlying cybersickness and the role of head movements, this study paves the way for future research and development efforts aimed at creating more immersive and comfortable VR experiences.

## Figures and Tables

**Table 1:** Comparison of VRSQ scores and head movement variables across different VR viewing conditions.

Variables		F-Value	*p*-Value	DVR	SVR	CON	Post Hoc Analysis
VRSQ		83.687	0.001	30.557 (10.078)	58.057 (6.364)	14.723 (3.851)	SVR > DVR, CON
HeadPosX (m)	Mean	0.420	0.683	0.022 (0.041)	0.003 (0.031)	0.009 (0.008)	-
	CV	1.040	0.433	0.063 (0.497)	−8.177 (14.247)	0.36 (0.46)	-
	Integral	0.459	0.662	2.92 (5.367)	0.371 (4.085)	0.524 (0.502)	-
HeadPosY (m)	Mean	1.048	0.431	0.961 (0.014)	0.948 (0.027)	0.931 (0.019)	-
	CV	20.000	0.008	0.009 (0.002)	0.005 (0.001)	0.002 (0.001)	DVR > SVR, CON
	Integral	687.221	<0.001	127.378 (1.163)	128.489 (4.011)	56.447 (1.182)	DVR, SVR > CON
HeadPosZ (m)	Mean	1.162	0.400	0.134 (0.253)	0.117 (0.243)	−0.024 (0.012)	-
	CV	1.105	0.415	−0.472 (0.489)	−0.175 (0.157)	−0.176 (0.072)	-
	Integral	0.932	0.465	17.459 (33.024)	15.741 (32.879)	−1.451 (0.716)	-
HeadOrientationW (rad)	Mean	1.472	0.332	0.96 (0.05)	0.965 (0.049)	0.997 (0.001)	-
	CV	2.817	0.172	0.012 (0.012)	0.001 (0.01)	0.01 (0.001)	-
	Integral	219.413	<0.001	127.339 (8.626)	130.886 (7.146)	60.427 (0.04)	DVR, SVR > CON
HeadOrientationX (rad)	Mean	42.498	0.002	0.055 (0.017)	−0.026 (0.017)	−0.066 (0.006)	DVR > SVR > CON
	CV	4.321	0.100	0.694 (0.101)	−1.238 (1.43)	−0.088 (0.042)	-
	Integral	29.582	0.004	7.219 (2.06)	−3.447 (2.301)	−3.99 (0.353)	DVR > SVR, CON
HeadOrientationY (rad)	Mean	0.692	0.552	−0.124 (0.281)	−0.067 (0.301)	0.047 (0.009)	-
	CV	0.456	0.663	−0.076 (0.301)	0.039 (0.04)	0.041 (0.02)	-
	Integral	0.475	0.653	−15.962 (36.959)	−9.029 (40.719)	2.817 (0.561)	-
HeadOrientationZ (rad)	Mean	0.277	0.771	0.023 (0.017)	0.016 (0.009)	0.017 (0.003)	-
	CV	5.413	0.073	2.212 (1.027)	0.662 (0.667)	0.076 (0.024)	-
	Integral	1.020	0.439	3.004 (2.244)	2.108 (1.153)	1.009 (0.217)	-
SpeedX (m/s)	Mean	125.826	<0.001	0.014 (0.002)	0.002 (0.001)	0.001 (0.0)	DVR > SVR, CON
	CV	23.187	0.006	1.741 (0.114)	1.795 (0.203)	0.9 (0.201)	DVR, SVR > CON
	Integral	122.800	0.001	1.661 (0.235)	0.212 (0.059)	0.046 (0.005)	DVR > SVR, CON
SpeedY (m/s)	Mean	8.180	0.039	0.007 (0.004)	0.001 (0.01)	0.01 (0.001)	DVR > SVR, CON
	CV	20.169	0.008	1.796 (0.147)	2.028 (0.386)	0.966 (0.179)	SVR > DVR, CON
	Integral	8.649	0.035	0.828 (0.445)	0.123 (0.01)	0.026 (0.006)	DVR > SVR, CON
SpeedZ (m/s)	Mean	6.901	0.050	0.01 (0.006)	0.002 (0.001)	0.001 (0.0)	-
	CV	11.347	0.022	1.649 (0.112)	1.763 (0.196)	1.027 (0.235)	SVR > DVR > CON
	Integral	7.457	0.045	1.166 (0.68)	0.196 (0.025)	0.041 (0.001)	DVR > SVR, CON

**Table 2:** Statistical analysis of significant variables including effect sizes and power analysis across different VR viewing conditions.

Variables	Effect Size (η^2^)	Power
VRSQ (score)	0.903	1.000
HeadPosY CV	0.689	0.986
HeadPos Integral	0.987	1.000
HeadOrientationW Integral	0.961	1.000
HeadOrientationX Mean	0.825	0.999
HeadOrientationX Integral	0.767	0.995
SpeedX Mean	0.933	1.000

η^2^: effect size using eta-squared values (η^2^ > 0.14 = large effect; η^2^ > 0.90 = very large effect); Power: post hoc statistical power analysis (1 − β; power > 0.80 indicates adequate sample size).

## Data Availability

The datasets generated and analyzed during the current study are available from the corresponding author on reasonable request.
